# High-Density Genetic Linkage Map Construction Using Whole-Genome Resequencing for Mapping QTLs of Resistance to *Aspergillus flavus* Infection in Peanut

**DOI:** 10.3389/fpls.2021.745408

**Published:** 2021-10-21

**Authors:** Yifei Jiang, Huaiyong Luo, Bolun Yu, Yingbin Ding, Yanping Kang, Li Huang, Xiaojing Zhou, Nian Liu, Weigang Chen, Jianbin Guo, Dongxin Huai, Yong Lei, Huifang Jiang, Liying Yan, Boshou Liao

**Affiliations:** Key Laboratory of Biology and Genetic Improvement of Oil Crops, Ministry of Agriculture, Oil Crops Research Institute of the Chinese Academy of Agricultural Sciences (CAAS), Wuhan, China

**Keywords:** peanut, *Aspergillus flavus*, resequencing, genetic linkage map, QTL

## Abstract

The cultivated peanut (*Arachis hypogaea* L.), which is rich in edible oil and protein, is widely planted around the world as an oil and cash crop. However, aflatoxin contamination seriously affects the quality safety of peanuts, hindering the development of the peanut industry and threatening the health of consumers. Breeding peanut varieties with resistance to *Aspergillus flavus* infection is important for the control of aflatoxin contamination, and understanding the genetic basis of resistance is vital to its genetic enhancement. In this study, we reported the quantitative trait locus (QTL) mapping of resistance to *A. flavus* infection of a well-known resistant variety, J11. A mapping population consisting of 200 recombinant inbred lines (RILs) was constructed by crossing a susceptible variety, Zhonghua 16, with J11. Through whole-genome resequencing, a genetic linkage map was constructed with 2,802 recombination bins and an average inter-bin distance of 0.58 cM. Combined with phenotypic data of an infection index in 4 consecutive years, six novel resistant QTLs with 5.03–10.87% phenotypic variances explained (PVE) were identified on chromosomes A05, A08, B01, B03, and B10. The favorable alleles of five QTLs were from J11, while that of one QTL was from Zhonghua 16. The combination of these favorable alleles significantly improved resistance to *A. flavus* infection. These results could contribute greatly to the understanding of the genetic basis of *A. flavus* resistance and could be meaningful in the improvement of further resistance in peanuts.

## Introduction

The cultivated peanut, also known as groundnut, is one of the most important oil crops and is widely cultivated in most tropical and sub-tropical regions. China, India, the USA, and Nigeria account for more than 90% of the cultivated area of this crop. In China, peanuts (*Arachis hypogaea* L.) are grown on 4.5 million ha with a total production of 17.52 million t (FAOSTAT, 2019).

Aflatoxin contamination, which is caused by a pathogen, namely, *Aspergillus flavus*, is a huge threat to the peanut industry and other important crops such as corn, rice, and wheat (Mahato et al., 2019; Womack et al., 2020). Contaminated agricultural products could endanger the health of human beings and animals (Pittet, 1998; Desjardins, 2003; Kew, 2013). Peanut pods and seeds could be infected in the soil during pre-harvest or during drying, storage, and transport stages after being harvested (Torres et al., 2014). Comprehensive prevention strategies including using bio-control agents, having good agricultural practices, and planting resistant varieties were suggested to control aflatoxin contamination in peanuts (Torres et al., 2014). The breeding of varieties with resistance to *A. flavus* infection has been one of the main objectives in peanut breeding programs and was recognized as the most cost-effective measure to solve the problem (Wang et al., 2016). However, only a few germplasms were found to possess the resistance, and the genetic bases of their resistance remain unclear. The breeding of novel varieties faces a tough challenge in peanuts. Thus, understanding the genetic pattern of resistance to *A. flavus* infection is vital to the enhancement of resistance in peanuts.

The Indian commercial peanut variety J11 is a well-known resistant germplasm to *A. flavus* infection, while the underlying genetic bases of its resistance remain largely unclear. It was first reported by Mehan and McDonald (1980) and confirmed in different environments by Mehan et al. (1981) and Nigam et al. (2009). J11 was also reported to be resistant to *A. parasiticus* infection (Kisyombe et al., 1985). The infection resistance of J11 was reported to be related to drought stress, pod maturity (Mehan et al., 1986), and seed coat integrity (Asis et al., 2005). Nayak et al. (2017) deployed an RNA-seq approach to understand the host–pathogen interaction, and they found 4,445 differentially expressed genes (DEGs) at four critical stages after inoculation in J11 and JL 24 (a susceptible genotype). Zhao et al. (2019) conducted both transcriptomic and proteomic analyses to reveal the changes that occurred during the infection of J11 by *A. flavus*, and 663 DEGs and 314 proteins were identified. However, the key genetic loci or genes responsible for the infection resistance of J11 were still unclear.

In recent years, quantitative trait locus (QTL) mapping was successfully used to identify QTLs with resistance to *A. flavus* infection from two newly identified resistant germplasms named ICG12625 (Yu et al., 2019) and Xinhuixiaoli (Khan et al., 2020). ICG12625 is a landrace from Ecuador, and Yu et al. (2019) constructed a recombinant inbred line (RIL) population from the cross between ICG12625 and a susceptible variety, Zhonghua 10. A genetic map with 1,219 SSR loci was constructed, and two QTLs were identified on chromosomes A03 and A10 with 7.96 and 12.16% phenotypic variation explained (PVE), respectively. On the other hand, Xinhuixiaoli is a Chinese landrace with resistance to *A. flavus* infection, and it was crossbred with a susceptible variety, Yueyou 92, to generate an RIL population (Khan et al., 2020). A single nucleotide polymorphism (SNP)-based genetic map was constructed by specific length-amplified fragment sequencing (SLAF-seq), and a major QTL on A03 with 18.11% PVE and a minor QTL with 4.4% PVE on B04 were identified.

In order to reveal the underlying genetic basis of the durable resistance to *A. flavus* infection of the well-known variety J11 at the QTL level, a susceptible variety, Zhonghua 16, was crossbred with J11 to develop an RIL population, upon which a high-quality genetic map was constructed by whole-genome resequencing. Additionally, QTLs associated with resistance to *A. flavus* infection were identified in four environments in the present study.

## Materials and Methods

### Plant Materials

Zhonghua 16 is a variety developed by the Oil Crops Research Institute of the Chinese Academy of Agricultural Science (OCRI-CAAS), Wuhan, China, and is susceptible to *A. flavus* infection. J11 is a variety introduced from the International Crop Research Institute for the Semi-Arid Tropics (ICRISAT), Hyderabad, India, and is resistant to *A. flavus* infection. Zhonghua 16 (the female parent) was crossbred with J11 (the male parent) to develop a mapping population containing 200 recombinant RILs using the single seed descent (SSD) method. The RIL population (F_8_) was used for genotyping and genetic map construction. Four generations (F_7_-F_10_) were planted in the experimental station of the OCRI-CAAS in Wuhan, China in 4 consecutive years (2017, 2018, 2019, and 2020, respectively). The field trials were conducted using a randomized complete block design with three replications following the method reported by Yu et al. (2019). These trials were designated as four environments, i.e., WH2017, WH2018, WH2019, and WH2020, respectively. After harvesting, the peanut pods were dried immediately until the moisture content of the seeds was 5–8%. Healthy and mature peanut seeds were artificially selected for further analysis.

### Phenotyping of Peanut Seed Resistance to *Aspergillus flavus* Infection and Data Analysis

The percent seed infection index (PSII) for each RIL and parents to the toxigenic *A. flavus* strain AF2202 was identified according to a previously reported method (Yu et al., 2019). Briefly, about 20 peanut seeds from each line were sterilized with 75% ethanol for 3 min. Then, they were rinsed with sterile distilled water three times and inoculated with 1 ml of a conidial suspension (2 × 10^6^ conidia/ml) of *A. flavus*. These seeds were cultured at 29 ± 1°C in the dark for 7 days to investigate resistance. Then, the resistance level to *A. flavus* infection of each seed was divided into four different grades, namely, (1) grade 0: seed surface without spore coverage; (2) grade 1: seed surface has <1/3 spore coverage; (3) grade 2: seed surface covered 1/3–2/3 spore; (4) grade 3: seed surface exceeds 2/3 spore coverage. The PSII was calculated according to the formula:


(1) $$\begin{array}{r} PSII=\frac{\text{n}1+\text{n}2\times 2+\text{n}3\times 3}{\text{N}\times 3}\times \;100\%, \end{array}$$

where n1, n2, and n3 represent the number of seeds of grade 1, grade 2, and grade 3, respectively, and N represents the total number. The SPSS 25 software [IBM Corporation] was used for the statistical analysis of PSII. The broad-sense heritability (*H*^2^) for PSII was calculated using the equation *H*^2^ = $\sigma_{\text{g}}^{2}$/($\sigma_{\text{g}}^{2}$ $+\sigma_{\text{gXe}}^{2}$/n$+\sigma_{\text{e}}^{2}$/rn), where $\sigma_{\text{g}}^{2}$ is the genetic variance component, $\sigma_{\text{gXe}}^{2}$ is the genotype-environment interaction variance component, $\sigma_{\text{e}}^{2}$ is the residual (error) variance component, and *n* and *r* were defined as the number of environments and replications, respectively.

### Library Construction and Sequencing

Young leaves were collected from the RIL population (F_8_) and used to extract genomic DNA using the cetyltrimethylammonium bromide (CTAB) method. The 300- to 500-bp Illumina Paired-end libraries were constructed for the 200 RILs and their parents. The PE150 reads for each library were generated by the Illumina HiSeq 2500 platform (Illumina, Inc., San Diego, CA, USA). The sequencing data have been deposited in the Sequence Read Archive database under accession number PRJNA760938. The raw sequencing reads were filtered by the Cutadapt (Marcel Martin Revision) and Trimmomatic software (Bolger et al., 2014) to generated high-quality clean reads. Then, the clean reads were aligned to the peanut reference genome (https://www.peanutbase.org/data/public/Arachis_hypogaea/Tifrunner.gnm1.KYV3/) using the BWA software (Li and Durbin, 2009). The HaplotypeCaller module of the GATK software (Broad Institute) was used to identify SNPs and InDels.

### Linkage Map Construction

Raw SNPs and InDels were filtered with the following criteria: (a) minor allele frequency (MAF) ≥ 0.2; (b) relative heterozygosity rate ≤ 0.2 (calculated according to Wu et al., 2016); (c) the proportion of missing genotypes ≤ 0.5; (d) polymorphism exists between parents. Then, the high-quality SNPs and InDels were used to construct a bin map using the MPR package (Xie et al., 2010). Linkage groups were named according to the locations of SNPs/InDels on the reference genome.

### QTL Analysis

The QTL mapping of the phenotypic data was performed using the default setting of the BIP (QTL mapping in bi-parental populations) approach in the IciMapping 4.2 software (Meng et al., 2015). The scanning step was set as 1 cM, and the logarithm of the odds (LOD) threshold was set as 2.5 to detect additive QTLs. The QTLs were named with the initial letter “*q*” followed by the trait name “*PSII*” and linkage group. An English lowercase letter was added if two or more QTLs were identified in the same linkage group. For instance, two QTLs for PSII were detected on LG A08, and then they were named *qPSIIA08.a* and *qPSIIA08.b*. If multiple QTLs overlapped on the same linkage group, they are considered to be a consistent QTL across environments and designated with the same name.

## Results

### Phenotypic Variation of Resistance to *Aspergillus flavus* Infection

**Figure 1:**
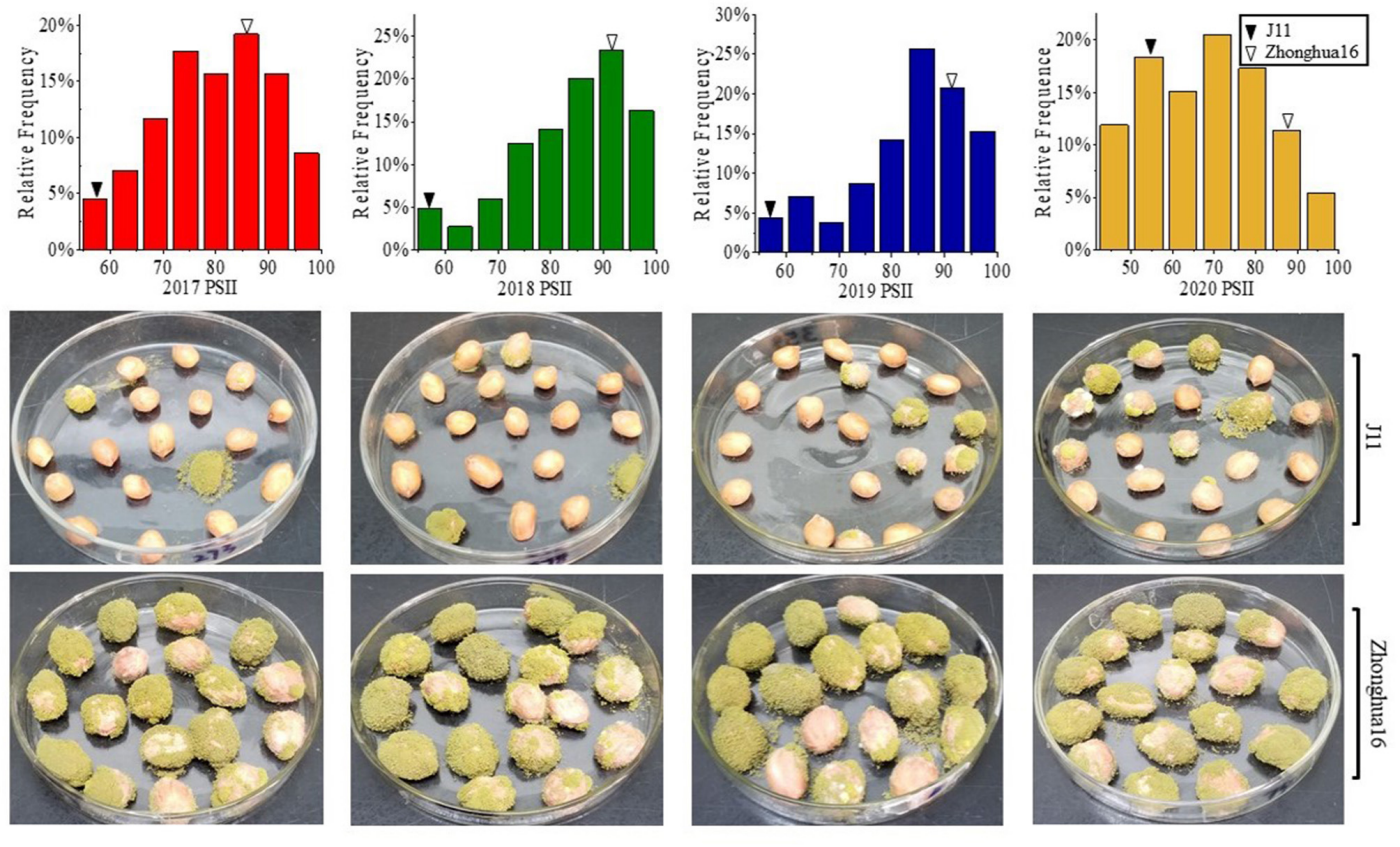
Phenotypic distribution of PSII in the RIL population and their parents harvested in Wuhan for 4 years. PSII, percent seed infection index.

The PSII of the RIL population was calculated with seeds harvested from 4 consecutive years (2017–2020). The PSII of the female parent Zhonghua 16 was significantly higher than the male parent J11. The PSII of Zhonghua 16 ranged from 87.04 to 93%, whereas J11 ranged from 51.04 to 55.97% in the four environments. The PSII varied among RILs from 46.92 to 98.25%, 40.74 to 100%, 26.98 to 100%, and 24.73 to 100% in the 4 years (Table 1). Continuous distributions with transgressive segregation were observed, which indicated that both parents contain resistant genes against *A. flavus* infection (Figure 1). The correlation coefficient of the PSII across the 4 years ranged from 0.374 to 0.96, which were significant at the *p* < 0.01 level (Supplementary Table 1). The results of an ANOVA for PSII showed significant differences among genotypes, environments, and genotypes × environments interactions at *P* < 0.001 (Table 2). The broad-sense heritability of the PSII was estimated to be 0.76, indicating that the PSII was mainly controlled by genetic factors.

**Table 1:** Phenotypic variation in PSII of the RIL population in Wuhan during 4 years.

**Env**	**Parents**		**RILs**		**CV**
	**Zhonghua 16**	**J11**	**Range**	**Mean ± SD**	
WH2017	87.04 ± 9.44	55.97 ± 8.57^*^	46.92–98.25	80.58 ± 10.57	0.13
WH2018	91.15 ± 6.26	53.83 ± 6.68^**^	40.74–100.00	83.74 ± 11.20	0.13
WH2019	93.00 ± 7.06	51.04 ± 6.70^**^	26.98–100.00	83.35 ± 11.55	0.14
WH2020	91.73 ± 5.80	51.65 ± 6.76^**^	24.73–100.00	67.87 ± 16.18	0.24

*PSII, percent seed infection index; Env, environment; WH, Wuhan, China; SD, standard deviation; CV, coefficient of variation;*

* *Difference is significant at p < 0.05 level between parents*;

** *Difference is significant at p < 0.01 level between parents*.

**Table 2:** Analysis of variance for the PSII of the RIL population across four environments.

**Source**	***df***	**Mean square**	***F*-value**	***P*-value**
Genotypes	199	0.08	8.71	<0.001
Environments	3	16.55	1776.57	<0.001
Genotypes × Environments	551	0.02	2.37	<0.001
Error	1,291	0.01		

*PSII, percent seed infection index*.

### Sequencing and Construction of Genetic Map

**Figure 2:**
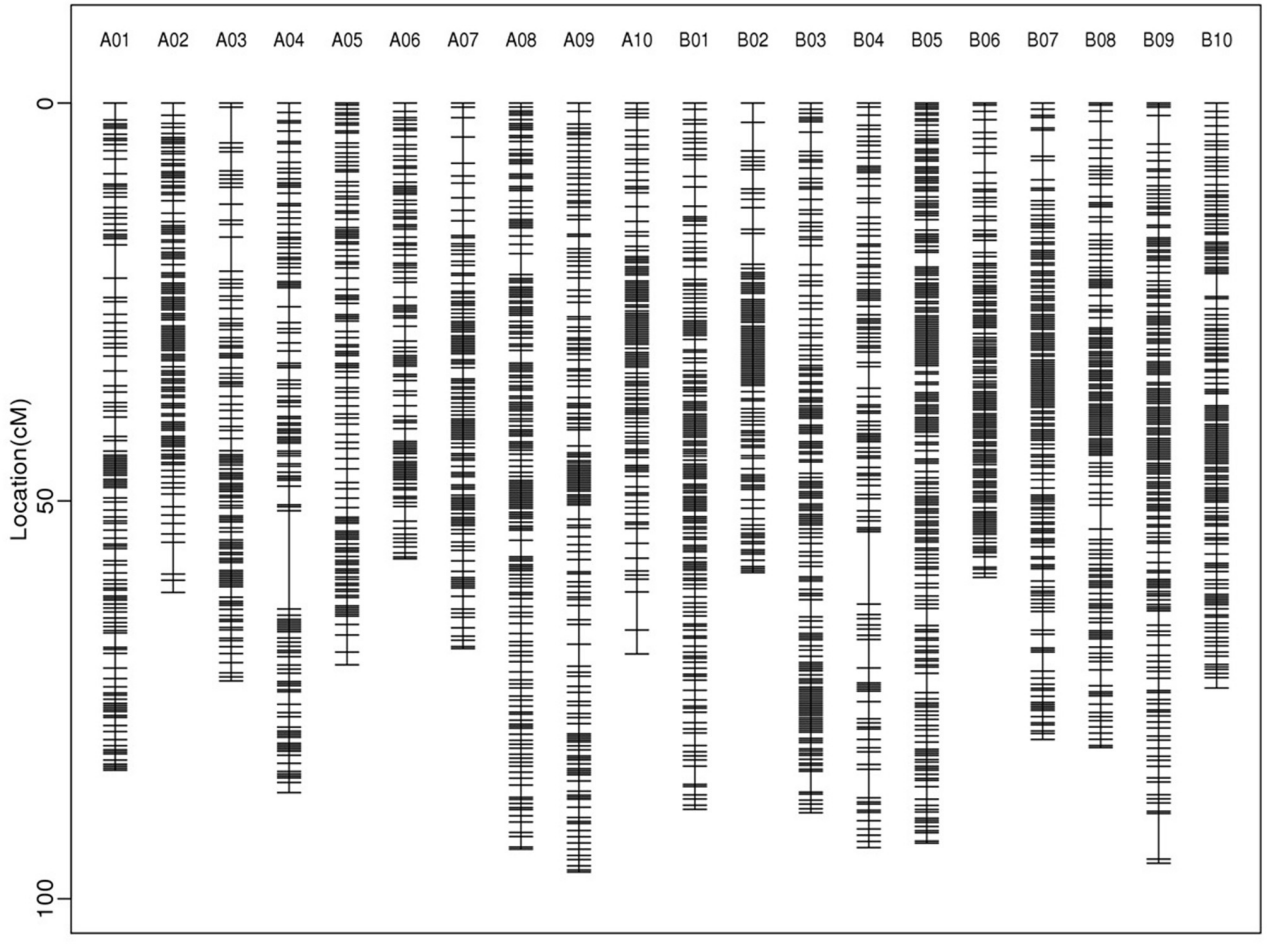
Distributions of bins on 20 linkage groups of the high-density genetic linkage map constructed in the RIL population.

About 5 billion reads were generated from the 200 RILs and their parents. The female parent Zhonghua 16 was sequenced at 8.95 × coverage and the male parent J11 at 8.35 × coverage, while the RIL population individuals were sequenced at ~2.96 × coverage (Supplementary Table 2). On average, 84.22% of reads were uniquely aligned to the reference genome (Supplementary Table 3). A total of 233,365 SNPs/InDels were used to construct a high-density genetic map. These markers were divided into 2,802 recombination bins, which generated the final genetic map covering 1573.85 cM with an average inter-bin interval of 0.58 cM (Table 3; Figure 2). There were 1,257 bins for A sub-genome with a map length of 760.69 cM, and 1,545 bins for B sub-genome with a map length of 813.16 cM. The length of LGs varied from 57.28 (A06) to 96.64 cM (A09), and the number of bins in LGs ranged from 102 (A06) to 210 (B05).

**Table 3:** Basic information of the high-density genetic linkage map of the RIL population.

**Chr**	**Length (cM)**	**No. markers**	**No. bins**	**Marker interval (cM)**	**Bin interval (cM)**	**Max interval (cM)**
A01	83.85	3,420	119	0.025	0.71	4.19
A02	61.50	13,895	124	0.004	0.50	3.99
A03	72.62	3,185	110	0.023	0.67	4.49
A04	86.66	2,042	120	0.042	0.73	12.34
A05	70.59	3,169	116	0.022	0.61	2.15
A06	57.28	2,409	102	0.024	0.57	1.87
A07	68.56	5,634	122	0.012	0.57	3.30
A08	93.76	5,298	181	0.018	0.52	2.63
A09	96.64	6,017	147	0.016	0.66	2.76
A10	69.23	15,859	116	0.004	0.60	4.80
B01	88.76	22,268	164	0.004	0.55	2.43
B02	59.01	14,568	110	0.004	0.54	3.88
B03	89.19	5,201	167	0.017	0.54	2.62
B04	93.57	3,352	116	0.028	0.81	9.10
B05	93.00	23,930	210	0.004	0.45	2.43
B06	59.62	21,306	143	0.003	0.42	2.43
B07	79.98	20,055	164	0.004	0.49	3.30
B08	80.99	17,578	148	0.005	0.55	3.00
B09	95.53	24,980	174	0.004	0.55	5.74
B10	73.51	19,199	149	0.004	0.50	2.91
Whole	1573.85	233,365	2,802	0.013	0.58	4.02

### Evaluation of Genetic Map

To evaluate the quality of the high-density genetic map, the sources of the bins in each RIL were analyzed, and the results showed that the bins from each parent form continuous fragments as expected (Supplementary Figure 1). The collinearity analysis, which compared the genetic position of all bins to their physical position on the reference genome, indicated a high collinearity between LGs and their corresponding chromosomes (Supplementary Figure 2).

### Detection of Additive QTLs for Resistance to *Aspergillus flavus* Infection

**Figure 3:**
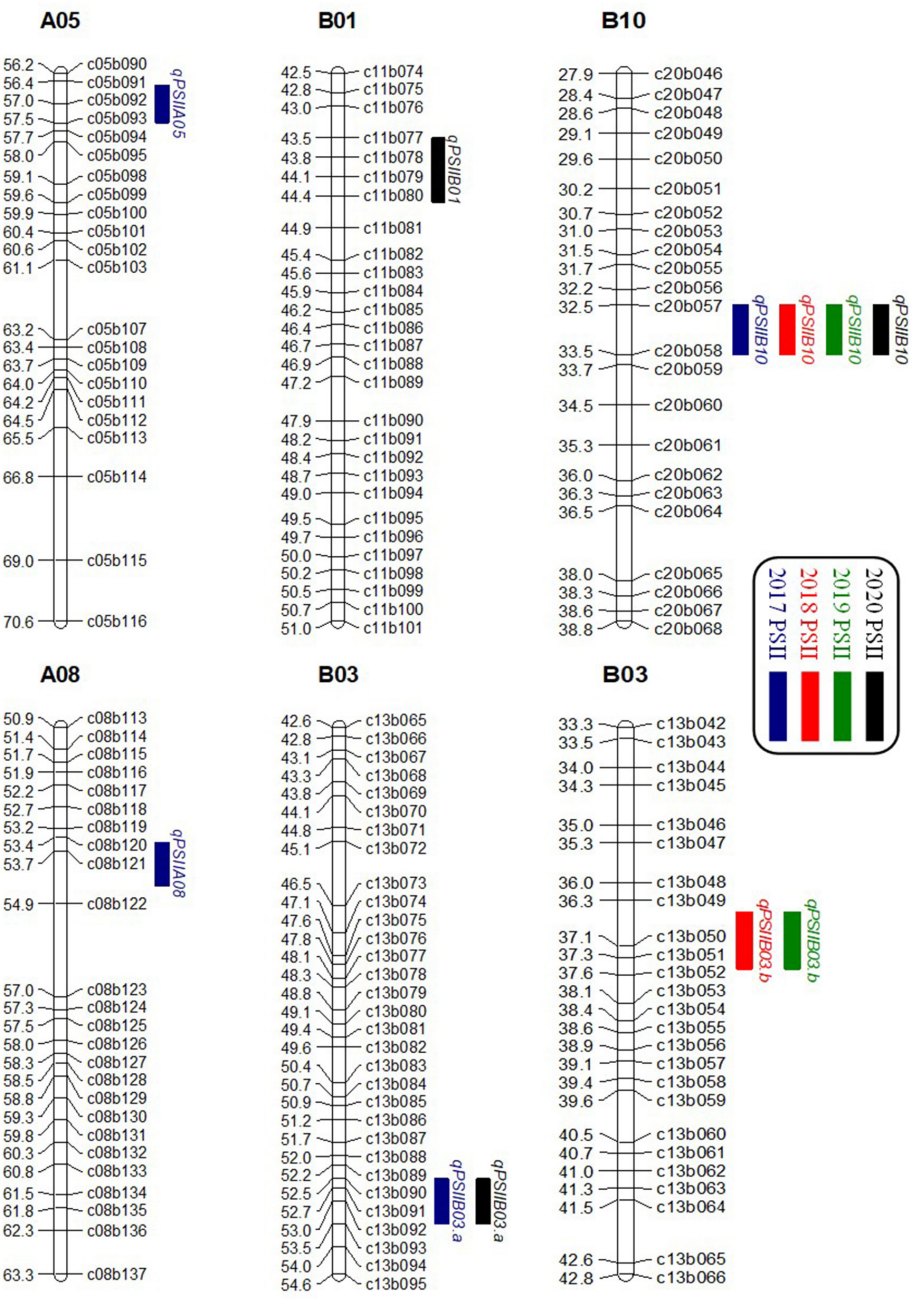
Distributions of QTLs for PSII on the genetic map in 4 consecutive years. PSII, percent seed infection index.

A genome-wide QTL analysis was performed using the high-density genetic map and the phenotypic data of the PSII from the 200 RILs in 4 consecutive years. A total of six additive QTLs were identified with 5.03–10.87% PVE (Table 4; Figure 3). In the WH2017, WH2018, WH2019, and WH2020 trials, four, two, two, and three QTLs could be identified and explained 35.63, 14.97, 16.33, and 22.3% total phenotypic variance, respectively. Their LOD values ranged from 2.63 to 5.97. Two QTLs were detected on B03, and the other four QTLs were on A05, A08, B01, and B10, respectively. The QTL *qPSIIB10* was consistently detected in 4 years, showing 6.91–10.58% PVE. Both QTL *qPSIIB03.a* and QTL *qPSIIB03.b* were repeatedly detected in 2 years with 9.16–9.23 and 5.03–5.75% PVE, respectivelyin. The major QTL *qPSIIA08* was only detected in 1 yearin with 10.87% PVE. In addition, the minor QTLs *qPSIIA05* andin *qPSIIB01* were only detected in 1 year.

**Table 4:** Additive QTLs for resistance to *Aspergillus flavus* infection in the RIL population across four environments.

**QTL**	**LG**	**Env**	**Cl (cM)**	**Marker interval**	**LOD**	**PVE (%)**	**Add**
*qPSIIA05*	A05	WH2017	56.5–57.5	c05b092–c05b093	3.17	5.50	2.41
*qPSIIA08*	A08	WH2017	53.5–54.5	c08b121–c08b122	5.97	10.87	3.39
*qPSIIB01*	B01	WH2020	43.5–44.5	c11b078–c11b079	2.63	6.16	3.74
*qPSIIB03.a*	B03	WH2017	52.5–53.5	c13b091–c13b092	5.16	9.16	3.07
		WH2020	52.5–53.5	c13b091–c13b092	3.91	9.23	4.58
*qPSIIB03.b*	B03	WH2018	36.5–37.5	c13b049–c13b050	2.74	5.03	2.68
		WH2019	36.5–37.5	c13b049–c13b050	2.75	5.75	2.87
*qPSIIB10*	B10	WH2017	32.5–33.5	c20b057–c20b058	5.39	10.10	−3.23
		WH2018	32.5–33.5	c20b057–c20b058	5.28	9.94	−3.79
		WH2019	32.5–33.5	c20b057–c20b058	4.92	10.58	−3.91
		WH2020	32.5–33.5	c20b057–c20b058	2.84	6.91	−3.99

*LG, linkage group; Cl, confidence interval of QTLs; PVE, phenotypic variance explained; Add, additive effect*.

### Phenotypic Effect of the Combinations of Resistant and Susceptible Alleles

The positive additive effects of *qPSIIA05, qPSIIA08, qPSIIB01, qPSIIB03.a*, and *qPSIIB03.b* indicated that the alleles from J11 confer resistance to infection. On the contrary, the negative additive effect of *qPSIIB10* showed that the alleles from Zhonghua 16 were responsible for improving resistance to infection. The recombination of *qPSIIA05, qPSIIA08, qPSIIB01, qPSIIB03.a, qPSIIB03.b*, and *qPSIIB10* were screened in the RIL population. As shown in Table 5 and Supplementary Table 4, RILs with the genotype R_1_R_2_R_3_R_4_R_5_R_6_ possessed all resistant alleles of the six QTLs from both parents, while those with the genotype S_1_S_2_S_3_S_4_S_5_S_6_ possessed all susceptible alleles of the six QTLs. The RILs with the genotypes R_1_R_2_R_3_R_4_R_5_S_6_ and S_1_S_2_S_3_S_4_S_5_R_6_ possessed resistant alleles from J11 and Zhonghua 16, respectively. Notably, the RILs with the genotype R_1_R_2_R_3_R_4_R_5_R_6_ showed significantly lower PSII than RILs with other genotypes (R_1_R_2_R_3_R_4_R_5_S_6_, S_1_S_2_S_3_S_4_S_5_R_6_, and S_1_S_2_S_3_S_4_S_5_S_6_), indicating that the combinations of resistant alleles could enhance resistance.

**Table 5:** Phenotypic effect of the combinations of resistant and susceptible alleles of the six QTLs in the RIL population.

**Genotype**	**No. of lines**	**WH2017 (%)**	**WH2018 (%)**	**WH2019 (%)**	**WH2020 (%)**
R_1_R_2_R_3_R_4_R_5_R_6_	5	55.53 ± 6.17^a^	64.40 ± 7.85^a^	63.91 ± 7.57^a^	42.61 ± 11.27^a^
R_1_R_2_R_3_R_4_R_5_S_6_	4	81.67 ± 9.66^b^	89.02 ± 5.50^b^	88.52 ± 4.09^b^	67.34 ± 7.98^b^
S_1_S_2_S_3_S_4_S_5_R_6_	5	90.38 ± 4.53^b^	91.23 ± 8.89^b^	94.23 ± 5.78^b^	84.98 ± 10.37^b^
S_1_S_2_S_3_S_4_S_5_S_6_	10	87.37 ± 6.63^b^	93.27 ± 5.37^b^	93.58 ± 5.27^b^	75.63 ± 6.84^b^

*“R_1_” to “R_5_” indicated the resistant allele from J11 for the top five QTLs in Table 4, while “R_6_” indicated the resistant allele from Zhonghua 16 for the last QTL qPSIIB10 in Table 4. “S_1_” to “S_5_” indicated the susceptible allele from Zhonghua 16 for the top five QTLs in Table 4, while “S_6_” indicated the susceptible allele from J11 for the last QTL qPSIIB10 in Table 4. The letters a and b after the phenotypic value were statistically different at p < 0.05 based on an ANOVA and Games–Howell multiple comparison*.

## Discussion

With the change of peanut harvest methods and the aggravation of global warming, the risk of peanut contamination by aflatoxin is also increasing. The most valid measure to control this disease is cultivating varieties with resistance to *A. flavus* attacks. However, the breeding of novel resistant varieties is still a challenge in peanuts, and high-yield resistant varieties were arduously unavailable in peanut production because the genetic mechanism of resistance to *A. flavus* is still unclear. In the present study, we analyzed the infection resistance of the RIL population derived from the internationally recognized peanut variety J11 with resistance to *A. flavus* in order to explore the quantitative genetic loci of resistance.

A high-quality genetic map is the fundamental basis of the QTL mapping of agronomic traits. Varshney et al. (2009) constructed the first map of the cultivated peanut, but this map only has 135 SSR markers. More genetic maps have been constructed with the development of abundant SSR markers. Luo et al. (2018) constructed a genetic map involving 830 SSR markers. However, the number of SSR markers is much lower than that of SNP markers in the genome. Relying on reduced-representation genome sequencing technologies, SNP markers were unearthed for the construction of a genetic map in peanuts. For example, Hu et al. (2018) built a genetic map containing 2,334 SNP/SSR markers and Liu N., et al. (2020) built a genetic map comprising 2,595 SNPs. Recently, two reports used whole-genome resequencing technology to develop genetic maps, containing 2,156 recombination loci (Agarwal et al., 2018) and 3,634 recombination bins (Liu H., et al., 2020), respectively. In the present study, we re-sequenced 200 RILs and both parents. upon which 233,365 SNPs/InDels were identified and formed 2,802 recombination bins on the genetic map. Compared with the previous maps, this map was high-density and would provide convenience for the mapping of resistance to *A. flavus* infection and other traits.

The QTL for the resistance of peanut seeds to *A. flavus* infection could explain the relatively low amount of total variance. Yu et al. (2019) screened the PSII of 140 RILs in two environments, and the identified QTLs explained 13 and 19.28% of the total phenotypic variation, respectively. Khan et al. (2020) evaluated the PSII of 208 RILs in two environments, and the total phenotypic variations explained by the identified QTLs were 20.81 and 24.19%, respectively. In the present study, QTLs were identified using 200 RILs in four environments, and the total phenotypic variations explained by identified QTLs were 35.63, 14.97, 16.33, and 22.3%, respectively. Therefore, the resistance of peanut seeds to *A. flavus* infection might be controlled by multiple small effect loci and influenced by the environment; thus, a significantly larger mapping population and a more accurate resistance phenotyping method should be used in future studies to map these QTLs. Forty years ago, the peanut variety J11 was discovered to be resistant to *A. flavus* infection (Mehan and McDonald, 1980). Subsequently, its stable infection resistance was proven in multiple pieces of research (Mehan et al., 1981, 1987; Nigam et al., 2009). However, this precious material has not been fully utilized, for the resistant loci or genes of J11 were not found. In the present study, the QTL mapping of its resistance was first studied using an RIL population. Specifically, six additive QTLs for resistance to *A. flavus* infection were identified on chromosomes A05, A08, B01, B03, and B10. These QTLs were novel because the previously reported QTLs by Yu et al. (2019) and Khan et al. (2020) were on A03, A10, and B04. Notably, the favorable alleles of five of the six novel QTLs were from J11, but these QTLs were not stable across environments. Three of them were only identified in one environment, and the other two QTLs were identified in two of four environments, indicating that the resistance to *A. flavus* infection of J11 might be controlled by multiple QTLs and was significantly influenced by growth environments. This phenomenon was consistent with the previous observation that drought stress, pod maturity (Mehan et al., 1986), and seed coat integrity (Asis et al., 2005) were related to the resistance of J11 to *A. flavus* infection. However, the RILs possessing the five resistant alleles from J11 had lower PSII than those with susceptible alleles but were not significant, indicating that the identified QTLs might be part of the genetic factors controlling resistance. Therefore, J11 could be crossed with more varieties with different backgrounds to construct more and larger populations to further dissect its resistance to *A. flavus* infection.

Surprisingly, the favorable alleles of the stable QTL *qPSIIB10* were from the susceptible parent Zhonghua 16, indicating that attention should also be paid to susceptible germplasms since they might possess valuable loci for resistance to *A. flavus* infection. Another important finding is that the RILs pyramided the favorable alleles of the six additive QTLs that significantly improved their resistance levels across all environments, although the effect of a single QTL was influenced by the environment. Therefore, both major QTLs and minor QTLs should be concerned with the genetic enhancement of resistance to *A. flavus* infection in peanut breeding.

## Conclusion

The present study constructed a high-density genetic map with 2,802 recombination bins based on whole-genome resequencing technology. The results identified six important QTLs for peanut seed resistance to *A. flavus* infection across four environments. A further analysis of QTL revealed that the accumulation of all favorable alleles of the six QTLs could significantly enhance the infection resistance, thus laying a foundation for further research on fine mapping and breeding application in peanuts.

## Data Availability Statement

The data presented in the study are deposited in the Sequence Read Archive repository, accession number PRJNA760938.

## Author Contributions

YJ, HL, YL, BL, and HJ conceived, designed, supervised the experiments, and contributed to the final editing of the manuscript. YL, BL, and HJ developed the RIL population. YJ, HL, BY, YD, JG, LH, XZ, WC, and NL conducted field trials and phenotyping. YJ, BY, YD, JG, and WC performed DNA extraction and genotyping. YJ, HL, XZ, BL, and HJ performed the data analysis and interpreted the results. YJ and HL prepared the first draft. All authors read and approved the final manuscript.

## Funding

This study was supported by the National Natural Science Foundations of China (Grant Numbers 31971903, 32001510, and 31761143005), the China Agriculture Research System (Grant Number CARS-13), the Crop Germplasm Resources Protection Project (Grant Number 2020NWB033), the Peanut Germplasm Resource Sharing Service Platform Project (Grant Number NCGRC-2020-036), and the Central Public-interest Scientific Institution Basal Research Fund (Grant Number 1610172019008).

## Conflict of Interest

The authors declare that the research was conducted in the absence of any commercial or financial relationships that could be construed as a potential conflict of interest.

## Publisher's Note

All claims expressed in this article are solely those of the authors and do not necessarily represent those of their affiliated organizations, or those of the publisher, the editors and the reviewers. Any product that may be evaluated in this article, or claim that may be made by its manufacturer, is not guaranteed or endorsed by the publisher.
