# Supplementary material for: High-Density Genetic Linkage Map Construction Using Whole-Genome Resequencing for Mapping QTLs of Resistance to Aspergillus flavus Infection in Peanut
Source: Front Plant Sci. 2021 Oct 21;12:745408. doi: 10.3389/fpls.2021.745408 (PMC8566722; doi:10.3389/fpls.2021.745408)
Supplement: Supplementary file 1 [file Data_Sheet_1.PDF]

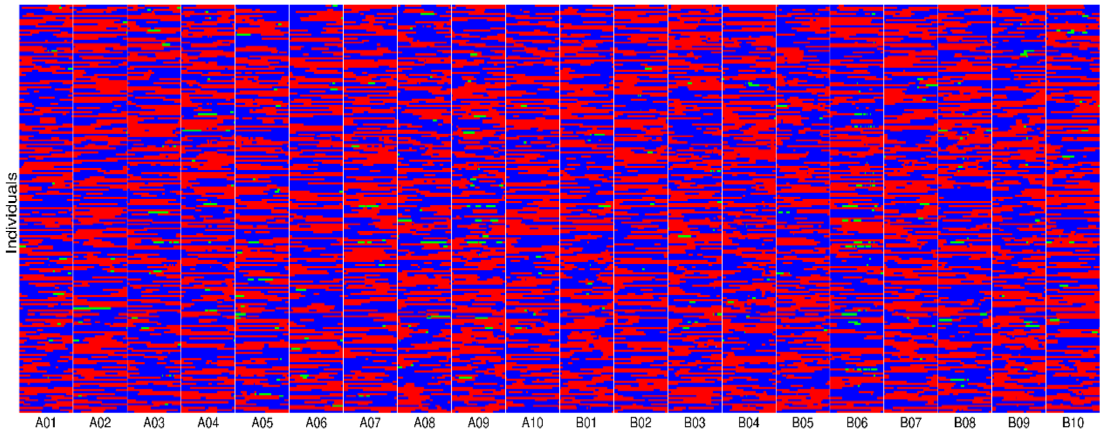

Fig. S1 Schematic diagram of the origin of recombination bins of the constructed genetic map. Blue: the genotypes of the recombination bins were from the susceptible parent Zhonghua16; Red: the genotypes of the recombination bins were from the resistant parent J11; Yellow: the genotypes of the recombination bins were heterozygous; RILs were arranged from the top to the bottom and chromosomes were arranged from left to right.
