# Supplementary material for: High-Density Genetic Linkage Map Construction Using Whole-Genome Resequencing for Mapping QTLs of Resistance to Aspergillus flavus Infection in Peanut
Source: Front Plant Sci. 2021 Oct 21;12:745408. doi: 10.3389/fpls.2021.745408 (PMC8566722; doi:10.3389/fpls.2021.745408)
Supplement: Supplementary file 3 [file Data_Sheet_3.PDF]

Table S1 Correlation analysis of PSII in RIL population

Environment	WH2017	WH2018	WH2019	WH2020
WH2017	1			
WH2018	0.601**	1		
WH2019	0.598**	0.960**	1	
WH2020	0.436**	0.374**	0.431**	1

**Correlation is significant at the $p < 0.01$ level
