# Supplementary material for: High-Density Genetic Linkage Map Construction Using Whole-Genome Resequencing for Mapping QTLs of Resistance to Aspergillus flavus Infection in Peanut
Source: Front Plant Sci. 2021 Oct 21;12:745408. doi: 10.3389/fpls.2021.745408 (PMC8566722; doi:10.3389/fpls.2021.745408)
Supplement: Supplementary file 4 [file Data_Sheet_4.PDF]

Table S2 The genome coverage and mapping depth of the RIL population and parents

Sample	Coverage(%)	Depth
QT1010	63.50	2.52
QT1011	60.10	2.31
QT1012	60.98	2.31
QT1013	65.74	2.54
QT1014	61.59	2.42
QT1015	59.69	2.30
QT1016	64.07	2.60
QT1017	65.68	2.67
QT1018	74.34	3.29
QT1019	63.88	2.55
QT1020	61.11	2.39
QT1021	65.72	2.63
QT1022	69.42	2.85
QT1023	69.80	2.88
QT1024	71.90	2.90
QT1025	71.72	2.69
QT1026	75.78	2.92
QT1027	71.66	2.84
QT1028	71.79	2.80
QT1029	70.51	2.70
QT1030	69.43	2.68
QT1031	76.76	3.30
QT1032	77.54	3.46
QT1033	73.59	2.87
QT1034	70.94	2.59
QT1035	69.27	2.51
QT1036	68.43	2.62
QT1037	71.71	2.86
QT1038	70.00	2.43
QT1039	69.13	2.70
QT1040	71.08	2.40
QT1041	69.46	2.81
QT1042	65.08	2.38
QT1043	70.47	2.67
QT1044	63.24	2.62
QT1045	85.09	4.34
QT1046	68.15	2.51
QT1047	70.49	2.69
QT1048	67.36	2.58
QT1049	83.30	4.90
QT1050	86.01	5.03
QT1051	76.86	3.30
QT1052	77.74	3.15
QT1053	76.38	3.38
QT1054	75.96	3.17
QT1055	76.23	3.29
QT1056	80.64	3.47
QT1057	75.47	3.15
QT1058	78.79	3.32
QT1059	66.76	2.55
QT1060	74.84	3.06
QT1061	73.50	3.49
QT1062	74.49	2.95
QT1063	69.02	2.55
QT1064	86.93	5.05
QT1065	76.45	3.23

QT1066	69.72	2.67
QT1067	76.77	3.10
QT1068	77.38	3.29
QT1069	71.63	2.64
QT1070	67.20	3.05
QT1071	61.77	2.58
QT1072	69.04	3.22
QT1073	70.87	3.42
QT1074	62.42	2.68
QT1075	69.04	3.07
QT1076	64.86	2.74
QT1077	65.36	2.96
QT1078	71.56	3.30
QT1079	79.99	5.85
QT1080	65.70	2.78
QT1081	63.34	2.74
QT1082	66.06	2.80
QT1083	67.40	2.77
QT1084	66.22	2.60
QT1085	66.72	3.00
QT1086	61.84	2.64
QT1087	62.75	2.67
QT1088	64.68	2.87
QT1089	64.01	2.80
QT1090	65.27	2.74
QT1091	72.59	3.30
QT1092	63.87	2.61
QT1093	66.32	2.83
QT1094	71.15	3.07
QT1095	67.50	2.66
QT1096	71.06	3.05
QT1097	69.81	2.83
QT1098	68.71	2.78
QT1099	69.98	2.85
QT1100	66.59	2.63
QT1101	70.97	3.09
QT1102	67.05	2.55
QT1103	69.50	3.05
QT1104	74.11	3.41
QT1105	68.74	2.85
QT1106	69.69	2.84
QT1107	70.58	3.12
QT1108	72.94	3.29
QT1109	68.36	2.70
QT1110	66.94	2.76
QT1111	74.03	3.42
QT1112	74.98	3.30
QT1113	66.81	2.76
QT1114	67.36	2.76
QT1115	66.39	2.72
QT1116	70.13	3.05
QT1117	64.08	2.55
QT1118	68.87	2.61
QT1119	73.75	3.05
QT1120	71.27	2.62
QT1121	71.77	2.74
QT1122	71.94	2.70
QT1123	68.40	2.56

QT1124	70.97	2.85
QT1125	70.39	2.64
QT1126	68.86	2.55
QT1127	69.89	2.77
QT1128	72.30	2.75
QT1129	69.69	2.62
QT1130	69.50	2.57
QT1131	71.37	2.74
QT1132	72.53	2.66
QT1133	73.31	2.75
QT1134	70.25	2.44
QT1135	71.36	2.70
QT1136	70.44	2.52
QT1137	68.94	2.63
QT1138	62.75	2.23
QT1139	70.49	2.51
QT1140	69.33	2.47
QT1141	76.78	3.59
QT1142	68.89	3.13
QT1143	65.59	2.96
QT1144	64.75	2.68
QT1145	67.96	3.17
QT1146	67.52	3.02
QT1147	72.66	3.46
QT1148	80.60	5.13
QT1149	67.31	3.03
QT1150	68.66	3.00
QT1151	68.58	2.91
QT1152	68.88	3.00
QT1153	69.46	3.13
QT1154	75.80	3.99
QT1155	66.84	2.95
QT1156	69.36	3.13
QT1157	55.12	2.79
QT1158	68.51	3.14
QT1159	63.00	2.63
QT1160	79.82	4.89
QT1161	67.37	3.04
QT1162	69.01	3.01
QT1163	63.01	2.70
QT1164	68.60	2.75
QT1165	68.59	3.15
QT1166	67.84	2.99
QT1167	64.08	2.76
QT1168	68.43	3.01
QT1169	81.34	6.00
QT1170	67.60	3.15
QT1171	64.00	2.80
QT1172	66.37	2.80
QT1173	65.50	2.86
QT1174	68.13	2.97
QT1175	63.60	2.67
QT1176	63.03	2.63
QT1177	73.96	4.37
QT1178	76.68	4.22
QT1179	63.05	2.73
QT1180	61.65	2.75
QT1181	61.24	3.10

QT1182	62.55	2.62
QT1183	58.96	2.55
QT1184	62.42	2.92
QT1185	66.75	3.04
QT1186	62.93	2.82
QT1187	67.20	3.02
QT1188	66.22	2.82
QT1189	62.26	2.64
QT1190	63.11	2.73
QT1191	61.75	2.53
QT1192	68.24	2.81
QT1193	64.68	2.68
QT1194	66.65	2.58
QT1195	68.59	2.92
QT1196	68.75	3.01
QT1197	67.23	3.57
QT1198	67.29	2.89
QT1199	64.25	2.63
QT1200	68.67	3.07
QT1201	67.29	2.89
QT1202	66.13	3.12
QT1203	65.44	2.78
QT1204	65.46	2.69
QT1205	66.46	2.88
QT1206	70.32	2.97
QT1207	69.06	2.86
QT1208	68.00	2.94
QT1209	63.41	2.57
Mean	69.08	2.96
J11	93.86	8.35
Zhonghua 16	92.35	8.95
