# Supplementary material for: High-Density Genetic Linkage Map Construction Using Whole-Genome Resequencing for Mapping QTLs of Resistance to Aspergillus flavus Infection in Peanut
Source: Front Plant Sci. 2021 Oct 21;12:745408. doi: 10.3389/fpls.2021.745408 (PMC8566722; doi:10.3389/fpls.2021.745408)
Supplement: Supplementary file 5 [file Data_Sheet_5.PDF]

Table S3 The sequencing reads and mapping rates for each RIL and parent

Sample	Total reads	No. uniquely mapped	Uniquely mapped
QT1010	22,930,348	18,868,292	82.29%
QT1011	20,134,740	16,692,717	82.91%
QT1012	20,682,383	16,851,121	81.48%
QT1013	22,863,335	18,881,968	82.59%
QT1014	22,200,073	18,132,555	81.68%
QT1015	19,894,413	16,351,700	82.19%
QT1016	24,587,081	20,167,664	82.03%
QT1017	26,078,829	20,903,275	80.15%
QT1018	35,475,349	29,462,416	83.05%
QT1019	23,623,223	19,434,735	82.27%
QT1020	21,487,359	17,349,782	80.74%
QT1021	24,772,158	20,433,225	82.48%
QT1022	22,189,442	18,930,070	85.31%
QT1023	22,364,994	19,075,158	85.29%
QT1024	23,002,775	19,789,130	86.03%
QT1025	21,617,308	18,326,233	84.78%
QT1026	24,283,240	20,966,088	86.34%
QT1027	23,076,728	19,613,450	84.99%
QT1028	22,226,130	19,070,192	85.80%
QT1029	20,927,866	18,098,969	86.48%
QT1030	20,955,293	17,755,232	84.73%
QT1031	29,697,458	25,252,119	85.03%
QT1032	30,804,947	26,483,302	85.97%
QT1033	24,751,034	20,970,429	84.73%
QT1034	21,296,496	18,078,771	84.89%
QT1035	20,152,372	17,215,803	85.43%
QT1036	20,846,066	17,903,155	85.88%
QT1037	23,615,404	20,214,522	85.60%
QT1038	19,797,124	16,841,938	85.07%
QT1039	21,385,474	18,313,335	85.63%
QT1040	19,010,881	16,637,783	87.52%
QT1041	22,384,147	19,135,735	85.49%
QT1042	18,147,279	15,329,485	84.47%
QT1043	21,646,747	18,536,743	85.63%
QT1044	19,590,007	16,484,691	84.15%
QT1045	41,920,614	36,051,563	86.00%
QT1046	19,987,881	16,997,598	85.04%
QT1047	21,647,141	18,615,179	85.99%
QT1048	20,162,246	17,262,448	85.62%
QT1049	48,449,082	41,057,076	84.74%
QT1050	51,282,430	43,474,899	84.78%
QT1051	29,733,613	25,583,208	86.04%
QT1052	28,166,866	24,132,360	85.68%
QT1053	29,862,603	25,275,028	84.64%
QT1054	28,232,306	23,792,525	84.27%
QT1055	29,421,807	24,722,997	84.03%
QT1056	32,079,688	27,240,832	84.92%
QT1057	27,880,839	23,501,533	84.29%
QT1058	31,022,098	25,943,672	83.63%
QT1059	20,013,717	16,881,788	84.35%
QT1060	26,386,223	22,508,325	85.30%
QT1061	30,306,967	25,480,012	84.07%
QT1062	25,577,746	21,738,205	84.99%
QT1063	21,048,916	17,438,299	82.85%
QT1064	50,807,472	43,676,655	85.97%
QT1065	28,247,451	24,158,938	85.53%
QT1066	22,161,039	18,836,901	85.00%
QT1067	29,277,705	24,106,056	82.34%
QT1068	29,704,658	25,062,166	84.37%
QT1069	22,078,080	18,820,986	85.25%
QT1070	23,783,179	20,106,408	84.54%
QT1071	18,700,025	15,791,912	84.45%
QT1072	26,367,091	22,000,814	83.44%

QT1073	28,640,332	24,031,471	83.91%
QT1074	19,702,303	16,614,010	84.33%
QT1075	25,376,522	21,118,093	83.22%
QT1076	21,812,447	18,266,950	83.75%
QT1077	22,613,615	18,916,108	83.65%
QT1078	27,565,230	23,070,498	83.69%
QT1079	54,713,160	45,744,572	83.61%
QT1080	20,861,464	17,809,001	85.37%
QT1081	20,645,343	17,185,607	83.24%
QT1082	21,982,207	18,229,796	82.93%
QT1083	21,710,204	18,068,309	83.22%
QT1084	19,953,714	16,875,616	84.57%
QT1085	23,964,008	19,816,632	82.69%
QT1086	19,567,842	16,126,217	82.41%
QT1087	20,275,819	16,627,762	82.01%
QT1088	22,600,550	18,651,910	82.53%
QT1089	20,945,559	17,507,195	83.58%
QT1090	20,859,342	17,633,963	84.54%
QT1091	28,522,706	23,942,208	83.94%
QT1092	19,710,142	16,534,704	83.89%
QT1093	22,340,937	18,736,518	83.87%
QT1094	24,389,128	20,812,339	85.33%
QT1095	20,264,711	17,033,006	84.05%
QT1096	24,226,058	20,403,315	84.22%
QT1097	22,242,388	18,795,715	84.50%
QT1098	21,457,185	18,152,580	84.60%
QT1099	22,486,046	18,941,235	84.24%
QT1100	20,104,408	16,655,129	82.84%
QT1101	24,304,820	20,591,004	84.72%
QT1102	19,324,215	16,175,306	83.70%
QT1103	23,477,713	19,949,557	84.97%
QT1104	28,024,794	23,777,777	84.85%
QT1105	22,131,844	18,954,819	85.65%
QT1106	22,513,960	19,253,633	85.52%
QT1107	25,224,134	21,398,551	84.83%
QT1108	27,504,282	23,360,175	84.93%
QT1109	21,037,685	17,865,014	84.92%
QT1110	20,754,308	17,767,666	85.61%
QT1111	29,002,161	24,632,962	84.93%
QT1112	29,009,052	24,346,542	83.93%
QT1113	21,191,622	17,888,541	84.41%
QT1114	21,296,300	18,035,474	84.69%
QT1115	20,645,303	17,495,706	84.74%
QT1116	26,316,995	21,558,462	81.92%
QT1117	20,168,436	16,482,560	81.72%
QT1118	20,817,158	17,528,297	84.20%
QT1119	25,671,859	22,031,924	85.82%
QT1120	20,698,659	18,179,133	87.83%
QT1121	22,641,985	19,149,080	84.57%
QT1122	21,994,146	19,012,805	86.44%
QT1123	20,614,042	17,430,843	84.56%
QT1124	22,764,063	19,793,439	86.95%
QT1125	21,261,273	18,202,837	85.61%
QT1126	20,172,437	17,168,778	85.11%
QT1127	22,892,582	19,322,216	84.40%
QT1128	22,573,844	19,413,453	86.00%
QT1129	20,373,574	17,667,048	86.72%
QT1130	20,019,146	17,164,210	85.74%
QT1131	22,454,351	19,208,601	85.55%
QT1132	21,624,331	18,793,238	86.91%
QT1133	22,835,250	19,788,698	86.66%
QT1134	19,228,301	16,506,824	85.85%
QT1135	21,792,744	18,882,275	86.64%
QT1136	20,005,726	17,255,115	86.25%
QT1137	20,383,775	17,512,453	85.91%

QT1138	15,740,093	13,600,742	86.41%
QT1139	20,026,938	17,357,018	86.67%
QT1140	19,666,675	17,105,757	86.98%
QT1141	31,797,535	27,202,512	85.55%
QT1142	25,662,104	21,572,271	84.06%
QT1143	23,117,986	19,047,104	82.39%
QT1144	20,587,229	17,232,368	83.70%
QT1145	25,222,481	21,177,371	83.96%
QT1146	24,310,816	20,084,067	82.61%
QT1147	29,955,315	25,436,645	84.92%
QT1148	49,124,493	40,904,334	83.27%
QT1149	23,744,309	19,978,116	84.14%
QT1150	24,234,383	20,377,026	84.08%
QT1151	22,875,000	19,498,164	85.24%
QT1152	23,689,176	19,965,304	84.28%
QT1153	25,587,062	21,381,715	83.56%
QT1154	35,014,306	29,844,219	85.23%
QT1155	22,924,888	19,243,846	83.94%
QT1156	25,591,071	21,354,797	83.45%
QT1157	20,027,085	15,654,705	78.17%
QT1158	24,934,854	20,955,526	84.04%
QT1159	19,401,534	16,242,452	83.72%
QT1160	46,200,855	38,686,629	83.74%
QT1161	24,182,972	20,080,612	83.04%
QT1162	24,324,339	20,703,565	85.11%
QT1163	20,468,920	17,087,817	83.48%
QT1164	21,393,832	18,327,015	85.66%
QT1165	25,874,428	21,624,768	83.58%
QT1166	24,181,334	20,137,570	83.28%
QT1167	20,844,220	17,616,283	84.51%
QT1168	24,519,860	20,506,722	83.63%
QT1169	58,487,410	48,661,729	83.20%
QT1170	24,861,938	20,784,566	83.60%
QT1171	23,772,535	19,830,033	83.42%
QT1172	24,306,527	20,686,096	85.11%
QT1173	22,307,527	18,584,567	83.31%
QT1174	23,742,630	19,991,710	84.20%
QT1175	19,673,418	16,705,568	84.91%
QT1176	19,408,522	16,403,982	84.52%
QT1177	40,126,484	32,783,130	81.70%
QT1178	40,358,028	33,252,689	82.39%
QT1179	19,880,981	16,899,919	85.01%
QT1180	19,996,572	16,797,582	84.00%
QT1181	23,685,803	19,235,847	81.21%
QT1182	19,475,693	16,315,947	83.78%
QT1183	18,255,291	15,059,884	82.50%
QT1184	23,184,726	18,708,627	80.69%
QT1185	24,467,758	20,244,329	82.74%
QT1186	21,198,508	17,792,984	83.94%
QT1187	24,680,283	20,580,403	83.39%
QT1188	22,435,738	18,738,512	83.52%
QT1189	20,121,709	16,718,764	83.09%
QT1190	21,135,952	17,501,369	82.80%
QT1191	18,956,610	15,691,489	82.78%
QT1192	22,031,229	18,463,213	83.80%
QT1193	20,368,223	17,041,241	83.67%
QT1194	20,305,658	16,929,243	83.37%
QT1195	23,208,797	19,596,247	84.43%
QT1196	23,994,534	20,192,503	84.15%
QT1197	29,455,253	23,680,747	80.40%
QT1198	22,513,888	18,901,962	83.96%
QT1199	19,427,452	16,455,982	84.70%
QT1200	24,550,691	20,579,477	83.82%
QT1201	23,051,254	19,066,761	82.71%
QT1202	24,113,303	20,104,062	83.37%

QT1203	21,305,058	17,833,527	83.71%
QT1204	20,521,218	17,255,052	84.08%
QT1205	22,523,234	18,598,261	82.57%
QT1206	24,073,214	20,273,945	84.22%
QT1207	23,254,671	19,466,852	83.71%
QT1208	24,208,201	19,877,989	82.11%
QT1209	19,390,971	16,038,539	82.71%
Zhonghua 16	106,379,137	90,253,809	84.84%
J11	100,042,369	85,111,072	85.08%
Mean	25,146,304	21,179,866	84.22%
