# Supplementary material for: High-Density Genetic Linkage Map Construction Using Whole-Genome Resequencing for Mapping QTLs of Resistance to Aspergillus flavus Infection in Peanut
Source: Front Plant Sci. 2021 Oct 21;12:745408. doi: 10.3389/fpls.2021.745408 (PMC8566722; doi:10.3389/fpls.2021.745408)
Supplement: Supplementary file 6 [file Data_Sheet_6.PDF]

Table S4 The detailed PSII of RILs used in the analysis of phenotypic effect of the combination of six QTLs

RIL	Genotype	PSII-2017(%)	PSII-2018(%)	PSII-2019(%)	PSII-2020(%)	Mean PSII(%)
QT1116	$R_1R_2R_3R_4R_5R_6$	53.33	63.33	62.75	35.69	53.78
QT1126	$R_1R_2R_3R_4R_5R_6$	.	72.55	68.52	56.25	65.77
QT1154	$R_1R_2R_3R_4R_5R_6$	63.65	57.89	56.12	24.73	50.60
QT1158	$R_1R_2R_3R_4R_5R_6$	46.92	55.83	58.29	45.13	51.54
QT1168	$R_1R_2R_3R_4R_5R_6$	58.21	74.07	75.93	51.25	64.87
QT1042	$R_1R_2R_3R_4R_5S_6$	85.00	82.35	83.33	64.93	78.90
QT1087	$R_1R_2R_3R_4R_5S_6$	92.34	95.83	93.34	70.21	87.93
QT1095	$R_1R_2R_3R_4R_5S_6$	65.99	.	.	56.14	61.07
QT1162	$R_1R_2R_3R_4R_5S_6$	83.33	88.89	88.89	78.07	84.79
QT1013	$S_1S_2S_3S_4S_5R_6$	89.34	98.33	96.30	97.69	95.42
QT1028	$S_1S_2S_3S_4S_5R_6$	98.25	76.67	.	91.19	88.70
QT1164	$S_1S_2S_3S_4S_5R_6$	87.03	98.25	98.15	76.85	90.07
QT1177	$S_1S_2S_3S_4S_5R_6$	85.31	97.92	98.15	69.27	87.66
QT1186	$S_1S_2S_3S_4S_5R_6$	91.99	85.00	84.31	89.90	87.80
QT1081	$S_1S_2S_3S_4S_5S_6$	78.57	96.67	97.44	84.74	89.36
QT1082	$S_1S_2S_3S_4S_5S_6$	90.68	94.12	95.56	81.38	90.44
QT1088	$S_1S_2S_3S_4S_5S_6$	80.18	78.95	80.50	77.01	79.16
QT1113	$S_1S_2S_3S_4S_5S_6$	88.86	94.74	93.75	75.12	88.12
QT1127	$S_1S_2S_3S_4S_5S_6$	85.36	96.67	96.97	77.22	89.06
QT1143	$S_1S_2S_3S_4S_5S_6$	87.69	94.44	95.02	75.41	88.14
QT1144	$S_1S_2S_3S_4S_5S_6$	94.87	91.23	.	80.72	88.94
QT1148	$S_1S_2S_3S_4S_5S_6$	93.77	100.00	98.55	76.58	92.22
QT1152	$S_1S_2S_3S_4S_5S_6$	76.94	91.11	89.18	69.21	81.61
QT1153	$S_1S_2S_3S_4S_5S_6$	96.73	94.74	95.24	58.91	86.41
